# Frailty and Age-Associated Assessments Associated with Chronic Kidney Disease and Transplantation Outcomes

**DOI:** 10.1155/2023/1510259

**Published:** 2023-04-01

**Authors:** Christian P. Fulinara, Alina Huynh, Deena Goldwater, Basmah Abdalla, Joanna Schaenman

**Affiliations:** ^1^Division of Infectious Diseases, Department of Medicine, David Geffen School of Medicine at UCLA, Los Angeles, CA 90095, USA; ^2^Divisions of Geriatrics and Cardiology, Department of Medicine, David Geffen School of Medicine at UCLA, Los Angeles, CA 90095, USA; ^3^Division of Nephrology, Department of Medicine, David Geffen School of Medicine at UCLA, Los Angeles, CA 90095, USA

## Abstract

**Background:**

Frailty is often defined as a decrease in physiological reserve and has been shown to be correlated with adverse health outcomes and mortality in the general population. This condition is highly prevalent in the chronic kidney disease (CKD) patient population as well as in kidney transplant (KT) recipients. Other age-associated changes include sarcopenia, nutrition, cognition, and depression. In assessing the contributions of these components to patient outcomes and their prevalence in the CKD and KT patient population, it can be determined how such variables may be associated with frailty and the extent to which they may impact the adverse outcomes an individual may experience.

**Objectives:**

We sought to perform a systematic literature review to review published data on frailty and associated age-associated syndromes in CKD and KT patients.

**Results:**

Over 80 references pertinent to frailty, sarcopenia, nutrition, cognition, or depression in patients with CKD or KT were identified. Systematic review was performed to evaluate the data supporting the use of the following approaches: Fried Frailty, Short Physical Performance Battery, Frailty Index, Sarcopenia Index, CT scan quantification of muscle mass, health-related quality of life, and assessment tools for nutrition, cognition, and depression.

**Conclusion:**

This report represents a comprehensive review of previously published research articles on this topic. The intersectionality between all these components in contributing to the patient's clinical status suggests a need for a multifaceted approach to developing comprehensive care and treatment for the CKD and KT population to improve outcomes before and after transplantation.

## 1. Introduction

Chronic kidney disease (CKD) is one of the leading causes of death in the US, affecting 1 in 7 adults, approximately 15% of the adult population [1]. According to the Centers for Disease Control and Prevention, CKD is most prevalent in adults aged 65 years and older, occurring at a rate of 38% [2]. In subsequent age groups, the incidence of CKD is 12% in those aged 45–64 years and 6% in those aged 18–44 years [2]. Therefore, CKD is a pervasive disease that disproportionately affects those in the geriatric population.

Given that CKD is the most common in the older adult population, it is important to understand the intersection between CKD and frailty. Frailty is defined as a decline in physical function and increased vulnerability to stressors as a result of a deterioration of physiological system(s) that can lead to adverse outcomes [3]. The presence of frailty is associated with the higher rates of mortality and hospitalization within the CKD population [3]. This review article characterizes the elements of frailty and other age-associated conditions as they relate to the patients diagnosed with CKD and who undergo kidney transplantation (KT) and the extent to which these elements affect patient outcomes. Additionally, it looks at posttransplant outcomes of those who received kidney transplants and how this relates to frailty.

## 2. Methodology

To find relevant articles pertaining to this review, online platforms such as PubMed and Google Scholar were used as primary sources when looking for articles. To guide collection of relevant research articles, primary search terms in combination with secondary search terms were used (Table 1).

The eligibility criteria for the inclusion of articles referenced are research articles that consisted of adult participants (over the age of 18) with CKD, not excluding articles that discussed CKD in conjunction with other comorbidities. An exclusion factor was for articles that discussed ongoing studies. Articles were also limited to those written in English or were readily translated to English, but may have focused on cohorts within non-English speaking countries. Articles were also selected that were published since 2009 to have the review article reflect the most recent findings. Additional references from selected articles were also included to support the relevant findings (Table 2). Conclusions from these findings are summarized in Table 3.

## 3. Results

### 3.1. Frailty-Associated Assessments for Individuals with CKD

Because of the association between frailty status and adverse outcomes, frailty assessments have been useful tools to evaluate the condition of a multitude of different patient populations and have also played a role in determining transplant eligibility.

There are a number of different frailty assessments that have been used to evaluate patients with CKD. Three of the most commonly used are Fried's Frailty Phenotype (FFP), Short Physical Performance Battery (SPPB), and the Frailty Index (FI). FFP evaluates individuals with a combination of subjective and objective measures including self-reported unintentional weight loss, self-reported exhaustion, weakness, gait speed, and physical activity [4, 43]. Another commonly used assessment is SPPB which measures physical performance through a series of functional tests (balance, chair stand, and gait speed) [7, 44]. SPPB, unlike FFP, does not rely on self-reported data and therefore provides a more objective alternative to quantify frailty [7, 66]. As opposed to FFP and SPPB, the FI captures frailty status through a “cumulative deficit” model, using the presence or absence of 70 electronic health record aging-related diagnoses to calculate the FI score [3, 10]. Using these methods, the prevalence of frailty, as measured using FFP in older adults with CKD, is as high as 21% among those 20 years and older to as high as 45%, among those older than 65 years old [22, 43]. Additionally, the risk of frailty, defined by FFP, increases with the greater severity of the kidney disease, as observed by data from the United States' third National Health and Nutrition Examination Survey of those with CKD [43]. Moreover, the presence of frailty, as defined by SPPB, is associated with multiple morbidities; subclusters within the CKD patient population that included CKD-related anemia, CKD-mineral bone disease, and diabetes were among those with patients having the lowest SPPB scores when compared to the geriatric population with other mobilities not limited to vision and hearing impairment, asthma, and depression, as observed by the United States' Screening of CKD among Older People across Europe study [44]. SPPB scores were also independently associated with severity of renal dysfunction within the Chronic Renal Insufficiency Cohort (CRIC), and thus, the severity of CKD is associated to poor physical performance [66].

Furthermore, patients with CKD who are frail, as determined by FFP, are much less likely to undergo transplantation as compared to their nonfrail counterparts. Adult candidates who were frail, based on FFP, from a three-center prospective cohort study among those on the kidney transplant waitlist had a 38% lower chance of being listed as opposed to nonfrail candidates [67]. Of those listed as potential transplant recipients, frail candidates were comprised of older individuals (55 ± 13 years), compared to the age range of nonfrail which was 52 ± 13 years [67]. When considering waitlist mortality, frail candidates had a 1.7 times higher risk [67]. Additionally, frail kidney transplant candidates were 35% less likely to undergo the transplantation procedure [67]. The patients' age did not affect the relationship between frailty time to listing nor waitlist mortality; therefore, it can be concluded that frailty is independently associated with increased risk of waitlist mortality and lower transplantation rate [67].

Aside from patient self-reported data and physical assessments, the presence of certain blood biomarkers may be indicative of one's frailty. Therefore, there have been advances in determining such biomarkers and evaluation of an inflammatory-frailty index [12]. Major inflammatory biomarkers studied alongside frailty consist of tumor necrosis factor alpha (TNF*α*), interleukin 6 (IL-6), and C-reactive protein (CRP) [12, 68, 69]. It is important to consider though that the inflammatory markers are not specific to frailty and are also influenced by the aging process and thus are considered alongside other modes to evaluate frailty [13]. Using FFP, we evaluated alongside a marker for inflammation to determine the predictive nature of the inflammatory-frailty index on the risk of mortality of kidney transplant candidates, frailty defined based on IL-6 increased mortality risk by 2.07-fold (95% CI: 1.03–4.19, *p* = 0.04) but did not improve posttransplant mortality risk in comparison to traditional FFP measurements [12]. Additionally, when considering IL-6 levels alone, there was a 1.91 increase in the likelihood of frailty with a 1 standard deviation change in log IL-6 levels (95% CI: 1.43–2.57) after adjusting for blood type, sex, age, and race [6]. Similarly, an increase in the odds of frailty was also observed in a 1 standard deviation change in CRP levels [6]. Conversely, more longitudinal studies had found no association between frailty and the CRP and IL-6 inflammatory markers [14]. When used in conjunction with frailty to predict mortality risk of kidney transplant recipients, IL-6 and CRP had a C-statistic of 0.777 and 0.728, respectively, as opposed to the mortality risk prediction of 0.646 when considering frailty alone [6]. Inflammatory markers, as well other biomarkers such as metabolites, immune markers, renal biochemistry, and genetic and epigenetic markers, have been shown to be potentially useful to assist with mortality risk prediction within the CKD population, but in predicting frailty alone, studies have offered conflicting results that require additional investigation to determine a more robust biomarker, or compilation of biomarkers, for frailty assessment [14, 68, 70–73].

Additional data regarding frailty's association with transplant patient outcomes follow in the section on posttransplant outcomes.

### 3.2. Additional Age-Associated Elements Associated with CKD

#### 3.2.1. Sarcopenia

Sarcopenia has no universally established definition but has been characterized as an age-related loss of muscle mass coinciding with a reduction of skeletal muscle function [76]. It is a common chronic geriatric condition among patients with CKD, especially in those in advanced stages [77, 78], and some authors predict that the prevalence of sarcopenia will increase by 72.4% in the total population by 2045, with prevalence in the elderly increasing from 11.1% to 12.9% [79]. Studies in CKD populations globally found that prevalence of sarcopenia ranged from 4 to 42% depending on the operational definition of sarcopenia used, stage of CKD, and population studied [78]. Independent of sarcopenic definition, severity of CKD increased the prevalence of sarcopenia [79]. When frailty was defined through modified criteria established by Fried et al., sarcopenia had been found associated with the condition in CKD patients [43]. It was found that sarcopenia and other metabolic conditions of CKD, such as anemia, acidosis, and vitamin D deficiency, strongly increased the odds of being diagnosed as frail [43].

The traditional method of evaluating for sarcopenia is quantification of muscle mass using imaging techniques such as computed tomography (CT); alternatively, the Sarcopenia Index (SI) is a much simpler method that can identify individuals at risk of adverse health outcomes and frailty [16, 18]. The SI quantifies sarcopenia by finding the ratio of creatinine to cystatin C serum levels, both markers of glomerular filtration rate (GFR) [16, 18]. Low SI levels are considered at risk for poor health outcomes, indicating less than sufficient skeletal muscle needed for creatinine production in proportion to a constant concentration of cystatin C [16, 18]. It was found that the SI was able to accurately predict muscle mass from abdominal CT scans [16, 18]. In a National Health and Nutrition Examination Survey (NHANES) from 1999 to 2006, it was found that using dual energy X-ray absorptiometry-derived appendicular lean mass index (ALMI, kg/m^2^) and fat mass index (FMI, kg/m^2^), both sarcopenia, defined by ALMI *T*-scores < −2, and relative sarcopenia, defined by fat-adjusted ALMI (ALMI_FMI_) *T*-score < −2, have been found to be associated with mortality both unadjusted (*p* < 0.01) and adjusted for variables such as sex, age, smoking status, status of other chronic conditions, education, and income (HR (sarcopenia): 2.20, 95% CI: 1.69–2.86; HR (relative sarcopenia): 1.60, 95% CI: 1.31–1.96) when defining CKD with creatinine and cystatin C serum levels [80]. In settings with more limited resources, it was found that using bioelectrical impedance analysis (BIA) to diagnose sarcopenia as reduced skeletal muscle mass (women: <6.76 kg/m^2^, men: <10.76 kg/m^2^) also showed that sarcopenia served as a predictor for mortality among those with CKD (HR = 2.89, 95% CI: 1.40–5.96, *p* = 0.004) [20]. However, it is also important to note the connection of frailty elements together, such as sarcopenia with nutrition. One study found that the prevalence of protein-energy wasting in sarcopenic CKD patients was higher than nonsarcopenic CKD patients (52 vs. 20%, *p* < 0.0001), indicating malnutrition without the presence of inflammation but with the presence of sarcopenia [81]. More on this continues in the next section.

#### 3.2.2. Nutrition/Malnutrition and the Microbiome

Nutritional status is a complication associated with frailty in geriatric CKD patients. When evaluating older patients with CKD, a FFP was shown to identify patients that could benefit from a comprehensive geriatric assessment (CGA) that includes a nutritional evaluation [22]. When defining malnutrition as the presence of protein-energy wasting (PEW) syndrome and/or a malnutrition inflammation score (MIS) > 7, frail CKD patients were malnourished through MIS (46% vs. 11%, *p* < 0.001) and had more PEW (38% vs. 21%, *p* = 0.047), compared to nonfrail CKD patients [22].

Among the geriatric nondialysis CKD population, patient-reported data for malnutrition illustrated that over a 4-year annual follow-up period, the average 7-point subjective global assessment (SGA) tool change was −0.18 points/year (95% CI: −0.21 – −0.14), indicating a decline in nutritional status [25]. This reduction was observed in more than one-third of participants, and 10.9% declined severely in SGA (≥2 points) [25]. Possible risks associated with this reduction included current smoking status, constipation and reduced appetite, and low physical component scores, which contribute to low physical activity, exhaustion, and weakness, which are attributes of frailty [25]. In another study performed among the same pool of possible subjects, protein-energy wasting (PEW) prevalence and its risk factors were evaluated, and it was found that reduction in SGA in conjunction with the presence of PEW was associated with muscle wasting, suggesting that nutrition should be started early among older CKD patients [26]. One study found a negative correlation between potassium intake (*r* = −0.410, *p* < 0.001) and CKD when measuring estimated dietary net endogenous acid production (eNEAP) that could affect kidney function, but no correlation was found with protein intake [82].

Loss of taste is also associated with CKD and has been shown to contribute to frailty and malnutrition [83]. Among a population of frail patients with CKD stages 3 or higher, it was found that lower probability of frailty was significantly associated with better objective function of taste (OR: 0.74, 95% CI: 0.57–0.97), better subjective function of taste (OR: 0.84, 95% CI: 0.74–0.96), and better intactness of the oral cavity (OR: 0.94, 95% CI: 0.9–0.98), independent of clinical features [83]. Such gustatory dysfunction influences nutritional status and appetite and can be developed due to ageusia, dysgeusia, or hypoxia as a result of impaired cognitive status, unmanaged electrolyte levels, or uremic toxins [83].

One interesting aspect of nutrition is its relation to the gut microbiome. The diversity of the microbiota has been shown to differ between healthy controls and those with renal conditions beyond CKD, which suggests a new avenue of research in regard to evaluating the role of nutritional elements like the gut microbiome in regard to CKD patients [84, 85]. More specifically, frailty among CKD patients has been found to be associated with malnutrition, likely related to the link between the gut microbiome and inflammation, a common characteristic of frailty [86]. CKD patients had a significantly higher abundance of bacteria in stool associated with pathological conditions such as vascular and inflammatory diseases (*Anaerotruncus*, *Citrobacter*, *Coprobacillus* genera, and *Ruminococcus torques* species) in comparison to non-CKD patients [86]. *Citrobacter* and *Ruminococcus torques* generate phenolic compounds such as p-cresyl sulphate (PCS) which has been found to accumulate in CKD patients [86]. When observing the gut microbiomes between frail and nonfrail CKD patients, it was found that *Coprobacillus*, *Anaerotruncus*, and *Dorea* genera had a higher abundance in frail patients compared to nonfrail patients in addition to *Coriobacteriaceae* family and *Eggerthella lenta* and *Eubacterium dolichum* species which have been shown in previous studies to be associated with frailty [86]. One pathway related to trimethylamine-*N*-oxide (TMAO), a gut microbial-dependent metabolite, was found to be elevated in CKD patients and, when found at high levels, was found to be predictive of mortality risk within 5 years (HR: 1.93, 95% CI: 1.13–3.29, *p* < 0.05) [87].

#### 3.2.3. Cognition

Cognitive decline is common among older adults; however, this decline is especially prevalent among frail individuals with CKD. Using tools such as the Mini Mental State Examination (MMSE) and Clock Drawing Test to assess cognitive status, one study found that frail adults with CKD have greater cognitive dysfunction compared to nonfrail individuals [22]. Moreover, all domains of cognition, especially in attention, recall, and function, are worse in patients with advanced CKD as compared to those without [89]. One study among patients with advanced CKD (eGFR <45) found that those with an eGFR <30 had the highest prevalence of severe cognitive impairment, when measured with the Modified Mini Mental State Examination (3MS) [30]. In a population with nonadvanced CKD, cognitive impairment was variable across the subject population but greatly increased across CKD stages [90]. It is likely that, in addition to frailty, cognitive decline could impact symptoms such as weakness, fatigue, and psychologic stress, possibly due to how cognitive impairment can contribute to reduced dependence on medication, resulting in faster progression [90]. Walking while talking has also been found to predict frailty and cognitive decline due to the possible presence of increased cognitive-motor interference among CKD patients, where it was found that those with CKD were slower and spent more time in stance (one foot in contact with the ground) and double-support (both feet in contact with the ground) gait phases, but when compared to walking alone, lower estimated GFR (eGFR) by 10 mL/min per 1.73 m^2^ was associated with a greater increase in time in the stance phase (95% CI: 0.2–1.5) [91]. In another NHANES study that looked at physical activity in regard to cognitive status, those with CKD stage G4-G5 had lower global cognitive function (difference = −0.38 SD, 95% CI: −0.62 – −0.15), but when accounting for physical activity, those in stage G4-G5 with high physical activity found no difference in cognitive function to those without CKD (difference = 0.10 SD, 95% CI: −0.29 – −0.49), unlike those with low physical activity with CKD stage G4-G5 in comparison to those without CKD (difference = −0.57 SD, 95% CI: −0.82 – −0.31) [92]. Cognitive function was tested with processing speed, verbal fluency, executive function, and immediate recall, where it was found that these were associated with CKD stage [92]. However, despite associations among cognitive status and CKD, it is important to note other comorbidities that can contribute to cognition. One study evaluating eGFR among CKD patients found that the odds of cognitive impairment increased by 47% among those with an eGFR <30 mL/min per 1.73 m^2^, in comparison to those with an eGFR equal to 45–59 mL/min per 1.73 m^2^ (odds ratio: 1.47, 95% CI: 1.05–2.05), when adjusted for traditional vascular risk factors, but not for hemoglobin, suggesting that anemia is an important marker for cognitive function in the CKD population [93].

Aside from comorbidities, immunosuppressive regimens may also have an effect on patients' cognitive function, most notably, processing speed and memory recall, a commonly tested cognitive task in frailty assessments [94]. Kidney transplant recipients can receive a wide variety of immunosuppressive drug therapy including tacrolimus or cyclosporine, mycophenolate sodium or mycophenolate mofetil, sirolimus, or steroids to reduce the risk of rejection of the transplanted organ [94, 95]. In a pilot study, there were observed differences due to immunosuppressive therapy from ANOVA, when controlled for age, months on dialysis, educational level, and time since transplantation, where patients that were put on tacrolimus or sirolimus performed significantly poorer on a direct and timed arithmetic task than those given cyclosporine and the healthy, nontreated control [94]. These results were also reflected in arithmetic tasks and inverse digits tests [94]. It is important to note that the failure for a kidney transplant to reverse cognitive decline may be due to early assessment after transplant due to high-dose immunosuppressants and associated early adverse events [95].

#### 3.2.4. Depression

Although not strictly part of a frailty assessment, depression is common in older adults. Within the CKD patient cohort, there is substantial overlap between frailty and depression, and higher frailty scores have been observed to be associated with higher depression scores in patients diagnosed with end-stage kidney disease [74, 75]. Aside from this association, the prevalence of depression has been shown not to be associated with eGFR and therefore does not vary with different stages of CKD [37]. The loss of certain aspects of physical functioning and increased incidences of frailty within the CKD patient population can trigger psychological changes that are illustrated by, but are not limited to, increased rates of depression and anxiety [96]. The presence of depression also impacts outcomes from having CKD, including decreasing motivation to adhere to treatment and increasing risk of hospitalization and mortality [35].

Individuals diagnosed with later stages of CKD may show an increasingly lower quality of life which may be a result of a greater prevalence of depression that negatively influences behavior toward treatment [38]. In individuals who have stage 3 or 4 CKD, the presence of depressive symptoms was found to be associated with poorer quality of life when evaluated at the 4^th^ year follow-up assessment [37]. Previous studies have also linked the presence of depressive symptoms and higher mortality risk: patients with advanced nondialysis-dependent CKD who were diagnosed with depression had a 6% higher mortality risk when adjusted for extraneous factors [97]. In terms of the prevalence of other comorbidities such as cardiovascular disease (CVD), higher rates of depression were observed in those with lower GFR and thus patients in the later stages of CKD (stages 4 and 5) [38].

In patients undergoing dialysis, the presence of depression, anxiety, stress, and psychological symptoms was associated with lower quality of life [96]. Surprisingly, years of dialysis and total Beck Depression Inventory-II (BDI-II) scores were found to be negatively associated; the more years a patient spent on dialysis, the fewer depressive symptoms experienced [42]. Other forms of therapy such as the cognitive behavior therapy that focuses on eliciting positive behavioral changes and encouraging emotional regulation have been illustrated to increase patients' adherence to dialysis, medication, and diet that ultimately improve the trajectory of their treatment [98]. In evaluating patients diagnosed with early stage of CKD (stages 1–3), depression was found to be linked to pain interference (measure of the extent pain impacts daily life), illness perception, and self-esteem, which are determining factors for depression [35]. When observed against depression, a significant positive association was observed with pain interference and illness perception, and a significant negative association was observed with self-esteem; therefore, there is a relationship between how CKD is perceived and the patient's perspective on their quality of life [35]. Additionally, in considering the strong associations with depression, intervention programs that directly address these risk factors can be incorporated into treatment to reduce adverse effects of CKD that are correlated with depression [35]. An example of health behavior intervention includes spiritual therapy which has proven to be effective in improving the overall self-esteem and self-efficacy of individuals undergoing hemodialysis; in incorporating health behavior interventions, it contributes to the aspect of holistic care [99].

### 3.3. Frailty Associations with Outcomes of Importance to Older Adults with CKD: Health-Related Quality of Life Assessment

According to Calman, quality of life (QOL) is a balance between desire and reality, namely, how closely the consequences of medical care align with an individual's expectation [42, 100]. Alongside the assessment of frailty among the patient population with CKD, a health-related quality of life (HRQOL) assessment can be used to aid in characterizing the wellbeing of the individual. The HRQOL assessment is comprised of questions pertaining to the physical functioning, emotional wellbeing, social function, and perception concerning general health to gauge self-perceived health status and social and emotional status [28]. One validated tool used in the CKD patient population is the RAND 36-Item Health Survey Version 1.0 (SF-36) [28].

In the geriatric population, it has been shown that frailty is associated with lower HRQOL, but the association between frailty and HRQOL is less well studied in the CKD patient population [28]. Analysis of FFP and RAND assessment illustrated a significant association between frailty and HRQOL [28]. Of the variables evaluated in the FFP, exhaustion had the most significant effect resulting in lower scores across all domains of SF-36 [28]. There was a strong correlation between the domains of HRQOL and Frailty Phenotype, when uncorrected and controlled for comorbidities, age, gender, and dialysis dependence [28]. Frailty was observed to have a significant association with SF-36 scores in various domains not limited to physical functioning, emotional problems, and social functioning, resulting in a 26-point lower score on the SF-36 for those categorized as frail [28].

Within the predialysis geriatric patient population in the early stages of CKD, a relation between depression and HRQOL has been shown. When controlling for comorbidities, age, gender, and eGFR, depression was independently associated with subcomponents of HRQOL; physical component summary (PCS) and mental component summary (MCS) were negatively correlated with GDS-15 (Geriatric Depression Scale-15) score [38].

Patients with end-stage kidney disease that undergo dialysis may experience psychological changes that induce depressive symptoms resulting in physical changes that ultimately affect their quality of life [42]. Variables of age and gender influenced patients' scores on the components of the mental QOL questions, most notably in the domain of social functioning in which case younger adults scored higher [42]. 21.4% and 14.3% of patients within the cohort had moderate to severe depressive symptoms, respectively, and this was also translated to lower scores in the mental portion of QOL [42]. Patients undergoing dialysis experienced an increase in mental QOL and a decrease in physical QOL over time [42]. Thus, mental health therapy for patients throughout their dialysis may be beneficial for decreasing their mortality risk and improving their quality of life [42].

#### 3.3.1. Predicting Posttransplant Outcomes

The severity of patient frailty can provide physicians an indicator of clinical outcomes after kidney transplantation [5, 6, 8, 45–48]. FFP was associated independently with a 2.17-fold higher risk of death after kidney transplant when adjusted for confounders and regardless of age (95% CI: 1.01–4.65, *p* = 0.047), as well as a longer length of stay (RR = 1.15, 95% CI: 1.03–1.29, and *p* = 0.01) and length of stay longer than 2 weeks (OR = 1.57, 95% CI: 1.06–2.33, and *p* = 0.03) when compared to nonfrail patients [49, 50]. In a similar study cohort, of the 5 FFP components, 2 combinations of these components increased risk of mortality after transplant: exhaustion with slower gait (HR = 2.43, 95% CI: 1.17–5.03) and exhaustion with slower gait and poor grip strength (HR = 2.61, 95% CI: 1.14–5.97) [51]. Another study found FFP to be an independent predictor of being at higher risk of early hospital readmission after adjusting for other risk factors (RR = 1.61, 95% CI: 1.18–2.19, and *p* = 0.002) [52]. Similar results have been found in regard to SPPB, where adult patients 5 years after transplant, who were considered impaired (SPPB ≤10) prior to admission, had 2.30-fold higher risk of posttransplant mortality than patients who were unimpaired (95% CI: 1.12–4.74, *p* = 0.02) [9]. This measure was further implicated on the finding that each one-point decrease in SPPB increased risk for posttransplant mortality 1.19-fold (95% CI: 1.09–1.30, *p* < 0.001) [9]. Other studies using SPPB have also found that frailty before transplant is independently associated to longer lengths of stay (relative time = 1.13; 95% CI: 1.05, 1.21, and *p* = 0.001) [53]. The cumulative deficit model that uses FI to define frailty has also been shown, when adjusted for age, sex, transplant type, and Social Vulnerability Index (SVI), to associate higher FI with increased chance of delisting and death among a retrospective cohort of adult solid organ transplant candidates which included heart, liver, and lung transplant candidates with kidney transplant candidates (HR: 1.03 per 0.01 FI score, 95% CI: 1.01–1.05, and *p* = 0.01) [11]. Defining frailty through chart review using the Frailty Risk Score (FRS) also has promise in predicting longer hospital lengths of stay and readmission after kidney transplant, where those with high FRS were, on average, readmitted 2.94 times, compared to those with low FRS, at 1.13 times (high FRS SD = 3.58, low FRS SD = 1.77; and CI: − 3.70–0.09) [54].

However, it is also important to note the dynamic nature of frailty [55]. Using FFP, a prospective cohort of kidney transplant recipients found that 22.0% of patients became more frail and 24.4% became less frail, when comparing frailty assessments during evaluation to assessment performed immediately prior to transplant [56]. Changes in frailty status were associated with the cause of end-stage renal disease and diabetes [56]. More importantly, in terms of posttransplant outcomes, mortality and length of stay after transplant were increased among those who became more frail after kidney transplant (mortality: 2.27-fold, length of stay: 2.02-fold) and among those who became more frail based on their original frailty score (mortality: 2.36-fold, length of stay: 1.92-fold) when compared to those who remained stable in their frailty status [56]. However, another study measuring long-term trajectories of frailty after kidney transplantation found that odds of frailty increased 2.5 years after transplant, despite odds decreasing immediately after transplant (OR = 1.03, 95% CI: 1.00 – 1.05) [57].

Age-associated elements with CKD have also been found to relate to frailty and/or posttransplant outcomes. One study found that one-fifth of patients who received kidney transplants became frail during the follow-up period after transplantation, where cognitive function, based on the Groningen Frailty Indicator (GFI), was most associated with a change in frailty (4.38, 95% CI: 0.59–32.24) [58]. It has also been shown that cognitive function improves in the short term in kidney transplant patients after transplant, but long-term results have shown that cognitive change rates differed between nonfrail and frail patients 1 year after transplant, and by 4 years after transplant, frail patients have lower cognitive scores overall [31]. Another study looking at the occurrence of sarcopenia among late-stage CKD transplant patients found that hospital readmission within 30 days after transplant was elevated among those with low muscle mass within 2 years prior to transplant (HR = 4.24, 95% CI: 1.40–12.90, *p* = 0.01) [17]. Recurrent falls among kidney transplant patients have also been found with increased mortality risk (HR = 51.43, 95% CI: 16.00–165.43), longer length of stay (RR = 1.13, 95% CI: 1.02–1.25), and graft loss within the first year (HR = 33.57, 95% CI: 11.24–100.21) [59].

Surgical complications among kidney transplant recipients typically fall within the categories of urological, vascular, and general, and those who were frail in some degree were found to more likely experience such complications (RR: 2.14; 95% CI: 1.01–4.54, and *p* = 0.035) [60]. Frailty is independently associated with delirium, delayed graft function, immunosuppression intolerance, and mortality after transplantation [60]. More specifically, a study investigating delirium claims in kidney transplant recipients from United States' registry claims, among the cohort, delirium incidence increased with age, where 20% of claims came from those who were frail and older than 75 years old [32]. It was found that delirium and frailty were independently associated (OR: 2.05, 95% CI: 1.02–4.13, and *p* = 0.04), but frailty was not directly associated with length of stay, institutional discharge, graft loss, and mortality, though these factors were associated with delirium alone [32]. Another study found that frailty identified before transplant was associated independently with increased risk for delayed graft function 1.94-fold (95% CI: 1.13–3.36, *p* = 0.02) [61]. However, frailty has not yet been proven to directly predict surgical complications [60]. In a pilot study of 18 kidney transplant candidates that underwent prehabilitation, there was an observed 64% improvement in physical activity level by the second month follow-up [62]. Considering these outcomes, multimodal prehabilitation might be a feasible route to improve physical functioning of patients prior to transplant [62].

It is also important to address the limitations of using frailty to understand posttransplant outcomes. One study on HRQOL scores among kidney transplant patients found that even though adult patients defined frail by FFP at admission had worse physical and kidney disease-specific HRQOL (*p* = 0.001); they were able to improve better than nonfrail patients in both physical (1.35 vs. 0.34 points/month, *p* *=* 0.02) and kidney disease-specific (3.75 vs. 2.41 points/month, *p* = 0.01) HRQOL, but faced no difference in mental HRQOL [63].

## 4. Discussion

**Figure 1:**
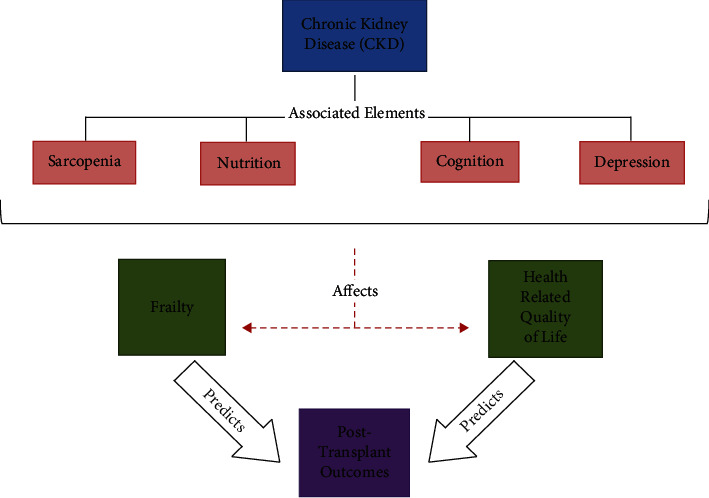
Chronic kidney disease (CKD) and its associated elements. Concept figure demonstrating interrelation between aging-associated dysfunction, frailty, qualify of life, and posttransplant outcomes.

The incidence of CKD can adversely impact an individual's quality of life and frailty status. Additional age-associated factors such as sarcopenia, nutrition, and depression can increase the risk of adverse outcomes and may potentially increase an individual's risk of mortality (Table 3). In this study, we have reviewed the various data supporting the various age-associated factors in both patients with CKD and in patients who have undergone KT in order to guide and educate clinicians and researchers regarding the available assessment approaches (Figure 1).

Frailty has been shown by several authors to be associated with CKD, occurring at higher incidences among this patient population. What remains unclear, however, is to what aspect of CKD drives this association, which may be related to uremia or other toxins not well cleared from the body in CKD patients vs. the impact of dialysis itself, which may have a negative impact on frailty. As the median age of patients with CKD continues to rise, it will be important to monitor whether the incidence of frailty increases in pre- and posttransplant patients, since the individual's frailty status can be a predictor of how long they will fare long term.

Sarcopenia is a prevalent condition within the CKD patient population. In accumulation with other metabolic conditions, it can increase an individual's risk of becoming frail, as well as risk of death [43, 80]. Sarcopenia and its association with death along with frailty should indicate a need to have an efficient way to properly diagnose the condition for CKD patients. Methods that are currently in use to diagnose the condition include the evaluation of muscle mass by CT, but such methods can be done routinely to monitor the trends in the patient's condition and trajectory of the disease. However, with continuing advances in understanding sarcopenia, possible use of the Sarcopenic Index could indicate an alternative and more accessible option to routinely assess for the condition (Table 2).

In terms of nutrition, malnutrition is associated with frailty and is a preventable condition, so it is important to identify the condition early on. With continuing research on the gut microbiome, it might also be helpful to examine its role and the extent the gut profile and inflammation contribute to an individual's nutrition and frailty status after having been diagnosed with CKD [84, 86]. Early interventions such as nutritional therapy with protein restriction and an energy adequate diet can help to curtail conditions such as hyperphosphatemia within the CKD population; additionally, physical exercise is advised to manage protein nutrition balance [101]. Regulating fluids is also vital within CKD patients as volume overload can contribute to the translocation of endotoxins and bacteria, affecting the intestinal flora and worsening the progression of the disease [101, 102].

Other elements such as cognition tend to decline with age, and specifically within the advanced CKD population, patients tend to have greater cognitive impairment and potentially increased cognitive-motor interference, with worsening CKD [90, 91]. Some studies are investigating how this cognitive impairment with frailty could indicate transplant outcomes [103]. Additionally, it is important to note the correlation between frailty and depression, especially within the later stages of CKD. The prevalence of depression impacts the individual's psychological state and can further affect their health-related quality of life [38]. Depression also has its influence on multiple aspects of CKD that may not be limited to treatment adherence but also illness perception, thus putting them at higher risk of hospitalization and mortality [35, 97]. The intersectionality between all of these components in contributing to the patient's frailty status suggests a need for a multifaceted approach to developing comprehensive care and treatment for the CKD population, especially regarding the various assessments that can be utilized to diagnose each of these elements (Table 2). Evaluating the quality of life of the patients takes into consideration both the physical functioning and emotional state, thus considering the impact of depression and frailty.

FFP and SPPB used to determine the patient's frailty status serve as a useful tool as a predictor of clinical outcomes after transplantation. Most notably, McAdams-DeMarco et al.'s study employed the use of FFP to determine the risk of posttransplant complication, finding an association between frailty and immunosuppression intolerance with higher mortality rate [49]. In addition to a higher risk of mortality, the occurrence of frailty increases the likelihood of early hospital readmission [49–52]. Frailty also increases the prospect that the patient experiences surgical complication and is further associated with delirium and graft loss and delayed graft function [32, 60]. Therefore, interventions that target the patient's frailty status, such as prehabilitation, might prove to be vital to minimizing adverse posttransplant outcomes by increasing the patient's physiologic reserves [49]. By routinely assessing these transplant candidates during regular office visits, regardless of age, providers are given additional insight into the condition of the patient and what interventions could be initiated to reduce their frailty or prefrailty status, if identified as such, as well as information that can be used in risk stratification. As mentioned earlier, various interventions could be initiated depending on the component of frailty that could bring about the most change upon a patient. In addition to implementing health interventions upon identifying frailty status, it is also possible to use this information as an indicator for suggesting additional resources for the patient that could be used both before and after transplantation.

Frailty within the CKD population is associated with multiple age-associated elements; moreover, the occurrence of frailty directly impacts the individual's health-related quality of life. In addition, frailty, despite its dynamic nature, has been found to be correlated with longer lengths of stay, early hospital readmissions, and delirium in addition to a higher risk of mortality among kidney transplant recipients [32, 49–52, 55]. Having a more comprehensive understanding of frailty can improve not only a patient's trajectory of the disease but also posttransplant outcomes and quality of life.

## 5. Conclusion

Chronic kidney disease, from many aspects, is correlated with increased health complications, lower quality of life, and increased risk of mortality. Frailty has been widely used to assess the likelihood of adverse outcomes at various CKD stages and in determining post-transplant outcomes. Given the many components contributing to frailty, it is important to have a defined categorization of the condition that would serve as a robust measurement across the various assessments to help in establishing frailty criteria. Although the relationship between frailty and CKD has been established, it may be useful to evaluate how frailty can be applied to assess patients at different stages including pre- and postdialysis initiation, and after transplantation. The intersectionality between various components in contributing to the patient's vulnerability to adverse events suggests a need for a multifaceted approach to developing comprehensive care and treatment for the CKD population. In treating patients early, by identifying what risk factors for frailty individual patients have, we may be able to avoid adverse posttransplant outcomes.

## Figures and Tables

**Table 1:** Primary and secondary search terms.

Primary	Secondary
“Chronic Kidney Disease”	“SPPB/FFP”
“Frailty”	“Cumulative Deficit”
“Frailty Assessment”	“Cognition”
“Kidney Transplantation”	“Cognitive Decline”
“Transplantation”	“Depression”
“Transplant”	“Nutrition/Malnutrition”
“Geriatrics”	“Diet”
“Risk Factors”	“Sarcopenia”
	“Hospitalization”
	“Length of Stay”
	“Mortality Rate”
	“Dialysis”
	“Hemodialysis”
	“Rehabilitation”
	“HRQOL”
	“Frailty Predict”
	“Biomarkers”

**Table 2:** Description of assessment types.

Assessment type	Purpose	Components	Scoring	Comments	References
*Frailty*					
Fried Frailty Phenotype (FFP)	Determine if an individual is frail, nonfrail, or at risk of becoming frail	Unintentional weight loss Poor endurance (self-reported exhaustion) Slowness (walking speed) Weakness (grip strength) Low physical activity level	Test score range: 0–5 Frail: ≥3 criteria present Prefrail: 1-2 criteria present Nonfrail: 0 criteria present	Access to dynamometer and stopwatch Walking space to measure gait speed	Fried et al., 2001 [4] McAdams-DeMarco et al., 2019 [5] McAdams-DeMarco et al., 2018 [6]
Short Physical Performance Battery (SPPB)	Determine an individual's frailty status	Gait speed Standing balance test Chair rise test	Test score range: 0–12 (each component scored 0–3) Frail: ≤9 Nonfrail: >9	Access to chair and stopwatch Walking space to measure gait speed	Bandinelli et al., 2006 [7] Chen et al., 2022 [8] Nastasi et al., 2018 [9]
Frailty Index (FI)/Deficit Accumulation Index (DAI)	Determine an individual's frailty status based on accumulation of deficits	Vary in components measured number of itemized variables. Typically, the index contains somewhere from 30 to 70 items.	Higher proportion of deficit present equates to a higher likelihood that individual will be frail	Variability in assessment types and number of variables Combined with other assessments such as the FI-CGA (frailty index combined with the comprehensive geriatric assessment)	Rockwood et al., 2007 [10] Varughese et al., 2021 [11]
Biomarker-Based Frailty Index	Determine an individual's frailty status based on biomarker blood concentrations	Evaluation of blood biomarker concentrations (inflammatory, immune, hormone)	Varies depending on biomarkers used and patient population	Cutoff values vary Biomarkers are a naturally part of aging and usage of this index would require coinciding with another frailty index	Haugen et al., 2021 [12] Mitnitski et al., 2015 [13] Baylis et al., 2013 [14]

*Sarcopenia*					
Computed tomography (CT)	To quantify muscle mass in a patient	4^th^ lumbar vertebrae abdominal CT scan to obtain paraspinal muscle surface area	Muscle mass was quantified by a skilled professional using an equation cited by Shen et al. [15]	Requires appropriate personal and financial resources Not feasible or practical for critically ill patients or busy treatment areas	Shen et al., 2004 [15] Kashani et al., 2017 [16] Wong et al., 2022 [17]
Sarcopenia Index (SI)	To provide an alternative method of quantifying muscle mass other than through CT or MRI	Serum creatinine to serum cystatin *C* ratio × 100	No definite ranges for patients; however, it is noted that SI is directly related to creatinine from muscles, so lower SI is suggested with reduced muscle mass	Cannot be applied to patients with acute kidney disease	Kashani et al., 2017 [16] Barreto et al., 2019 [18]
Bioelectrical impedance analysis (BIA)	To quantify muscle mass through the resistance of current run through the body	BIA analyzer and electrodes	Muscle mass was calculated by an equation cited by Janssen et al. [19]	Validity depends on hydration status	Janssen et al., 2000 [19] Pereira et al., 2015 [20]

*Nutrition*					
Comprehensive geriatric assessment (CGA)	To assess an elderly frail person's psychosocial, medical, and functional ability in order to develop a long-term care plan	Intensity of assessment can vary, where the most intense can include admission into a medical facility, where the patient completes the assessment and receives limited treatment from an interdisciplinary team	Varies	Can be costly and time intensive	Rubenstein et al., 1991 [21] Vettoretti et al., 2020 [22]
Malnutrition inflammation score (MIS)	To assess malnutrition and inflammation among dialysis patients to evaluate clinical outcomes	Contains 10 components with 4 levels of intensity within each component. The lowest score is 0 and highest score is 3 for each component	>7 is considered is malnourished Maximum score is 30	May not correlate completely with other laboratory and clinical measurements	Kalantar-Zadeh et al., 2001 [23] Vettoretti et al., 2020 [22]
7-point subjective global assessment (SGA) tool	Evaluates nutritional status based on standardized values	Considers 4 domains: weight change history, physical examination for fat loss, history of diet and gastrointestinal issues, and muscle wasting	<3 is considered severely malnourished Maximum score is 7	High intr-observer reliability suggests follow-up assessments	Visser et al., 1999 [24] Windahl et al., 2021 [25] Windahl et al., 2018 [26]

*Cognition*					
Mini Mental State Examination (MMSE)	To examine one's mental state, more specifically the cognitive aspects of mental functions	11-question measure used to test 5 domains of cognition: recall, language, registration, orientation, and calculation and attention	≤23 is indicative of cognitive impairment Maximum score is 30	Does not equal a diagnosis for a mental condition	Folstein et al., 1975 [27] Vettoretti et al., 2020 [22] Nixon et al., 2020 [28]
Modified Mini Mental State (3MS)	Improved MMSE	Additional test items to test for a larger variety of one's functional cognition	The maximum score is now 100	More sensitivity compared to MMSE	Teng and Chui, 1987 [29] Burns et al., 2018 [30] Chu et al., 2019 [31] Haugen et al., 2018 [32]

*Depression*					
Beck Depression Inventory-II (BDI-II)	Determines the presence and severity of depression within an individual	21-item inventory that includes sadness, self-criticalness, loss of pleasure, and guilty feelings	Total score range: 0–63 (each item scored 0–3) Nondepressed: 0–12 Dysphoric: 13–19 Dysphoric or depressed: 20–63	Scores may be affected by social desirability bias	Dozois et al., 1998 [33] García-Batista et al., 2018 [34] Duan et al., 2021 [35]
GDS-15 (Geriatric Depression Scale-15)	Screens for the presence of depression within the elderly population	15-item assessment consisting of questions pertaining to self and self in relation to others answered in a “yes/no” format	Total score range: 0–15 (each question scored 0 or 1) Normal: 0–4 Mild depression: 5–9 Moderate to severe depression: 10–15	Decreased completion rate with decreased cognitive function	Conradsson et al., 2013 [36] Feng et al., 2013 [37] Wang et al., 2019 [38]

*Health-related quality of life (HRQOL)*					
RAND 36-Item Health Survey Version 1.0 (SF-36)	Determine the health status of the population of interest and evaluate the effect of healthcare interventions	36-item assessment that covers 8 health concepts: physical functioning, bodily pain, role limitations due to physical health, role limitations due to emotional problems, emotional wellbeing, social functioning, energy/fatigue, general health perception	Assessment scored 0 (worst health) to 100 (best health)	Accounts for the variation in the number of chronic illnesses Assessment may be limited to be taken by individuals with higher cognitive and physical functioning	Hays et al., 1993 [39] VanderZee et al., 1996 [40] Andresen 1999 [41] Muflih et al., 2021 [42]

**Table 3:** Takeaways from this review and recommended areas of future research.

Conclusions	References	Potential research directions/considerations
Physical frailty testing such as SPPB and FFP can predict the posttransplant outcomes of individuals diagnosed with CKD.	[3–5, 7–9, 11, 17, 22, 31, 32, 43–65]	(i) Determine the robustness of each frailty metric in comparison to each other (ii) Determine the ideal timing of frailty assessment and how frailty changes with transplantation (iii) Determine the impact of physical therapy interventions on frailty and postoperative outcomes
Cumulative deficit assessment can identify patients at risk for adverse outcomes after kidney transplantation.	[3, 10, 11, 54]	(i) Determine what frailty testing approach is most effective in predicting posttransplant outcomes in patients with CKD
Whether a physical, cumulative deficit, or biomarker approach to frailty is more efficient and effective remains unknown.	[3, 6, 12–14, 66–75]	(i) Assessment of different frailty metrics either combined or separately to determine the ideal approach (ii) Identify the role each metric would play in the development/improvement in multidisciplinary care programs (iii) Evaluate frailty in other transplant populations to determine how frailty may be similar or different in nonkidney transplant candidates
Prevalence of sarcopenia is increased in patients with CKD compared with the general population.	[16, 18, 20, 43, 76–81]	(i) Determine the robustness of different evaluation methods for sarcopenia (ii) Identify the most beneficial intervention to reduce the impact of sarcopenia as well as other CKD-related conditions
Nutritional status is a controllable contributor to frailty.	[22, 25, 26, 81–88]	(i) Independent evaluations of nutrition in the prevalence of frailty (ii) Identify the effects of early action nutritional programs/interventions among the CKD population, including but not limited to personally curated diets and vitamin and probiotic supplementation (iii) Define gut microbiome patterns of frail patients
Cognitive impairment is more common in patients with CKD compared with the general population.	[22, 30, 89–95]	(i) Identify how cognitive decline can contribute to other independent frailty elements (ii) Determine whether interventions to improve cognition pretransplantation can impact postoperative delirium
CKD patient populations have an overall lower HRQOL and greater prevalence of depressive symptoms.	[28, 35, 37, 38, 42, 74, 75, 96–100]	(i) Development of mental health therapy interventions that address variables more affected in the HRQOL due to the onset of CKD (ii) Identify a system of metrics that would be used to measure the effectiveness of different forms of therapy on HRQOL and depressive symptoms (iii) Determine a form of mental health therapy that is the most effective for specific patient populations

## Data Availability

No independent data were used to support this study.
